# Online group psychotherapy for patients with binge eating disorder during COVID-19 emergency

**DOI:** 10.1192/j.eurpsy.2021.703

**Published:** 2021-08-13

**Authors:** F. Focà, F.R. Santucci, S. Ferretti, L. Rinaldi, G. Sani, L. Janiri, D.P.R. Chieffo

**Affiliations:** Istituto Di Psichiatria E Psicologia, Fondazione Policlinico Universitario A. Gemelli - IRCCS, roma, Italy

**Keywords:** binge eating disorder, online group psychotherapy, COVID-19 emergency, online psychotherapy

## Abstract

**Introduction:**

On March 13th 2020, in execution of the Law Decree 14/2020 regarding the reorganization of National Health Care related to COVID-19 emergency, all non-urgent outpatient healthcare services were suspended in Italy. The present work describes remote support and online group psychotherapy set in motion during COVID-19 emergency for outpatients with Binge Eating Disorder.

**Objectives:**

Aim of the present work is to describe and evaluate online support and group psychotherapy for outpatients with Binge Eating Disorder during lockdown due to COVID-19 emergency. Outcomes were evaluated by remote administration of questionnaires.

**Methods:**

20 outpatients with Binge Eating Disorder, treated by psychotherapists of Hospital Psychology Unit in Psychiatry Day Hospital of an Italian General Hospital, received remote support by phone calls and online group psychotherapy from march to may 2020. During the first two weeks, patients were supported via phone calls. From the third week on, they took part to online group psychotherapy sessions, held every week at the same day and time. Pre-post remote administration of Clinical Outcomes in Routine Evaluation-Outcome Measure (CORE-OM) and the Questionnaire of Eating Behaviours (Scheda dei Comportamenti Alimentari, SCA) was used to evaluate outcomes. Data were analyzed by Student’s t-test.

**Results:**

No significant difference was found, thus indicating stability of symptomatology.

**Conclusions:**

Lockdown was a highly stressful period, in which many people lost control on eating behaviours and those with Binge Eating Disorder were expected to have an exacerbation of symptoms. Remote support and online group psychotherapy proved effective in protecting patients from a possible aggravation of their condition.

